# 3D-Printed Polymer-Infiltrated Ceramic Network with Antibacterial Biobased Silver Nanoparticles

**DOI:** 10.1021/acsabm.2c00509

**Published:** 2022-09-27

**Authors:** Ľudmila Hodásová, A. Gala Morena, Tzanko Tzanov, Gemma Fargas, Luis Llanes, Carlos Alemán, Elaine Armelin

**Affiliations:** † Departament d’Enginyeria Química, IMEM-BRT, EEBE, Universitat Politécnica de Catalunya, C/Eduard Maristany, 10-14, Ed. I, 2nd Floor, 08019 Barcelona, Spain; ‡ Barcelona Research Center in Multiscale Science and Engineering, Universitat Politécnica de Catalunya, C/Eduard Maristany, 10-14, Basement S-1, 08019 Barcelona, Spain; § Grup de Biotecnologia Molecular i Industrial, Department of Chemical Engineering, Universitat Politécnica de Catalunya, Terrassa 08222, Spain; ∥ Departament de Ciéncia i Enginyeria de Materials, CIEFMA, EEBE, Universitat Politécnica de Catalunya, Campus Diagonal Besòs, C/Eduard Maristany, 10-14, Building I, 1st Floor, 08019 Barcelona, Spain; ⊥ Institute for Bioengineering of Catalonia (IBEC), The Barcelona Institute of Science and Technology, Baldiri Reixac 10-12, 08028 Barcelona, Spain

**Keywords:** polymer-infiltrated ceramic network, polyacrylates, lignin, laccase enzyme, silver nanoparticles, antibacterial activity

## Abstract

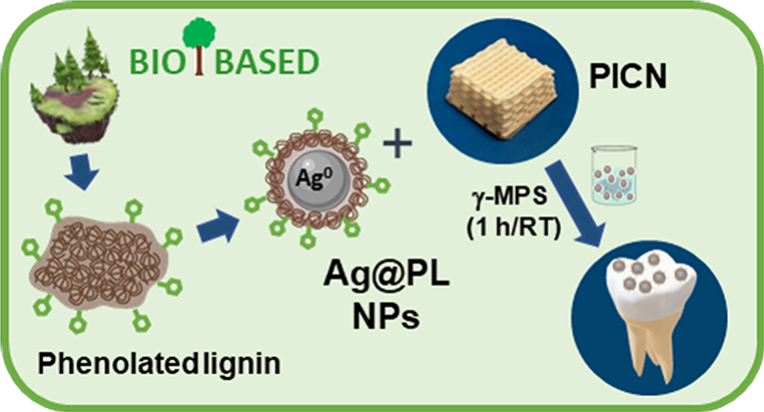

This work aimed at the antimicrobial functionalization of 3D-printed polymer-infiltrated biomimetic ceramic networks (PICN). The antimicrobial properties of the polymer-ceramic composites were achieved by coating them with human- and environmentally safe silver nanoparticles trapped in a phenolated lignin matrix (Ag@PL NPs). Lignin was enzymatically phenolated and used as a biobased reducing agent to obtain stable Ag@PL NPs, which were then formulated in a silane (γ-MPS) solution and deposited to the PICN surface. The presence of the NPs and their proper attachment to the surface were analyzed with spectroscopic methods (FTIR and Raman) and X-ray photoelectron spectroscopy (XPS). Homogeneous distribution of 13.4 ± 3.2 nm NPs was observed in the transmission electron microscopy (TEM) images. The functionalized samples were tested against Gram-positive (*Staphylococcus aureus*) and Gram-negative (*Pseudomonas aeruginosa*) bacteria, validating their antimicrobial efficiency in 24 h. The bacterial reduction of *S. aureus* was 90% in comparison with the pristine surface of PICN. To confirm that the Ag-functionalized PICN scaffold is a safe material to be used in the biomedical field, its biocompatibility was demonstrated with human fibroblast (BJ-5ta) and keratinocyte (HaCaT) cells, which was higher than 80% in both cell lines.

## 1 Introduction

In recent years, yttria-doped zirconia has gained a lot of attention as yttrium oxide (Y_2_O_3_) prevents crack propagation in sintered zirconia ceramics.^1−3^ However, there are still certain drawbacks of the material that prevent its use as a one-piece biomedical prosthesis, like in dental implants, which are composed of titanium screws, polymeric adhesives, and ceramic crown parts.^4,5^ The most important concerns are related to the high brittleness and high Young’s modulus of zirconia, which is incompatible with that of alveolar bones,^6^ and also its high surface roughness and porosity,^7^ which are ideal for bacteria growth if compared to titanium implants,^8^ for example. In our recent works, we have successfully combined a biocompatible adhesive copolymer with 3D-printed yttria-stabilized tetragonal zirconia scaffolds (3Y-TZP) with 50% infilled macropores^9^ to palliate crack propagation in 3D-printed polymer-infiltrated ceramic network (PICN) scaffolds under compression forces.^9,10^ Moreover, the hybrid materials conserve their biocompatibility, promoting the growth and proliferation of MG-63 osteoblast cells on their surface.

The developed PICN was inspired by the natural composition of teeth, comprised of inorganic and organic components.^11,12^ The infiltration of polyacrylate adhesives in a macroporous ceramic 3D-printed material was expected to prolong the lifespan of the implant since the polymer adhesive corrects the brittleness problem of the ceramic material.^13,14^ Improvement of 3D-printing techniques has made the design and production fast and easy, providing products of high-end quality.^15−20^ The main advantage is that the design of the pore size and distribution can be controlled with CAD/CAM processes and therefore adjusted according to the necessity of the application,^9,10^ which is not possible using traditional sintering methods of compact ceramic structures.^21^

The PICN sample itself does not apparently promote the growth of bacteria but does not have antimicrobial properties usually desirable in the biomedical field to prevent biofilm formation.^22^ Bacterial infections are a continuous risk to human health, primarily with the alarming increase of multidrug-resistant bacteria. An important percentage of these infections are acquired at healthcare facilities (e.g., hospitals and nursing homes).^23^ The incidence of biofilm formation in biomedical implants and devices is a great concern due to the difficulty in treating both the infection and the resulting surgery complications. In fact, bacterial adhesion and subsequent biofilm formation are the major causes of their failure. Thus, there is an urgent need to develop alternative antimicrobial devices, prostheses, and implants to face healthcare-associated infections.

Considering the wide spectrum of antibacterial properties of silver, it has become one of the most popular antibacterial agents. However, in a long term, the devices containing silver can release Ag^+^ ions, which might have cytotoxic effects. Silver nanoparticles (AgNPs) receive significant attention as the form of nanoparticles exhibits much higher reactivity in comparison with bulk material,^24,25^ which is a great advantage in treating bacterial infections. AgNPs release metal ions that cause changes in the membrane permeability^26^ and/or induce oxidative stress,^27^ leading to cell death. In addition, metal ions catalyze reactions that produce reactive oxygen species (ROS), causing oxidation of important cell structures like lipids and DNA.^28,29^

To decrease the cytotoxicity associated with metals, different biocompatible natural polymers have been used to produce hybrid metal-polymer NPs.^25^ For instance, chitosan was used to produce biocompatible hybrid Ag@chitosan NPs that effectively killed the Gram-positive and Gram-negative bacteria.^30^

Lignin gains sizeable attention as a renewable resource for production of low molar mass compounds or value-added materials.^31,32^ However, the processability is usually limited due to the low reactivity of lignin. Many investigations have been made to improve the reactivity of lignin, such as methylation (hydroxymethylation), demethylation, amination, and phenolation. The phenolation of lignin is commonly achieved by a chemical method in which lignin is treated with phenol under acidic conditions, leading to the condensation of phenol with lignin side chains.^33^ Recently, the green phenolation of lignin was achieved enzymatically using the laccase/mediator system.^34^ The highly reactive phenolated lignin (PL) can be used as a reducing agent for metals to synthesize metal NPs in an environmentally friendly route.^35^

In this work, we propose the use of biobased silver phenolated lignin nanoparticles (Ag@PL NPs) to impart antimicrobial activity for ceramic materials with 3D-printed PICN scaffold architecture. Such a hybrid material (ceramic and acrylate polymer adhesive) is used in dentistry applications.^36−38^ The nonshedding surfaces of crowns, teeth, fixed partial dentures, or endosseous implants facilitate the formation of thick biofilms.^39^ Due to the high surface tension of the methacrylate copolymer adhered to the zirconia platforms, antimicrobial nanoparticle adsorption by the dip-coating process does not work properly. Thus, the surface of PICN samples has been activated with Ag@PL NPs with the help of chemical etching and silane solution adhesion promoters. Therefore, covalent bonds have been achieved with the sol–gel technology, employing 3-(trimethoxysilyl)propyl methacrylate (γ-MPS) as an anchoring molecule, and stable antimicrobial PICN scaffolds were obtained for the first time.

## 2 Experimental Procedure

### 2.1 Materials

The 3Y-TZP (3 mol % yttria-stabilized tetragonal zirconia polycrystal) powder was supplied by SEPR Saint-Gobain ZirPro (France) under the commercial name CY3Z-R. The Protobind 6000 sulfur-free lignin (*M*_w_ = 1000 g·mol^–1^) used in this work was supplied by Green Value (Switzerland). The Pluronic F-127 hydrogel, γ-MPS (3-(trimethoxysilyl) propyl methacrylate), Bis-GMA (bisphenol A glycerolate dimethacrylate), TEGDMA (triethylene glycol dimethacrylate), BPO (benzoyl peroxide, Luperox A75), gallic acid, tannic acid, 3′,5′-dimethoxy-4′-hydroxyacetophenone (acetosyringone), silver nitrate, phosphate-buffered saline (PBS), Nutrient Broth (NB), and Dulbecco’s modified Eagle’s medium (DMEM) were all purchased from Sigma-Aldrich. AlamarBlue cell viability reagent was purchased from Invitrogen, Life Technologies Corporation (Spain). Laccase enzyme from *Myceliophthora thermophila* (Novozym 51003, Novozymes, Denmark) with an enzymatic activity of 1322 U·mL^–1^ was used. Two bacterial strains *Staphylococcus aureus* (*S. aureus*; ATCC 25923) and *Pseudomonas aeruginosa* (*P. aeruginosa*; ATCC 10145) and human fibroblast (ATCC-CRL-4001, BJ-5ta) and keratinocyte (HaCaT cell line) cells were received from the American Type Culture Collection (ATCC LGC Standards, Spain).

### 2.2 Synthesis of Silver Phenolated Lignin Nanoparticles (Ag@PL NPs)

**Figure 1:**
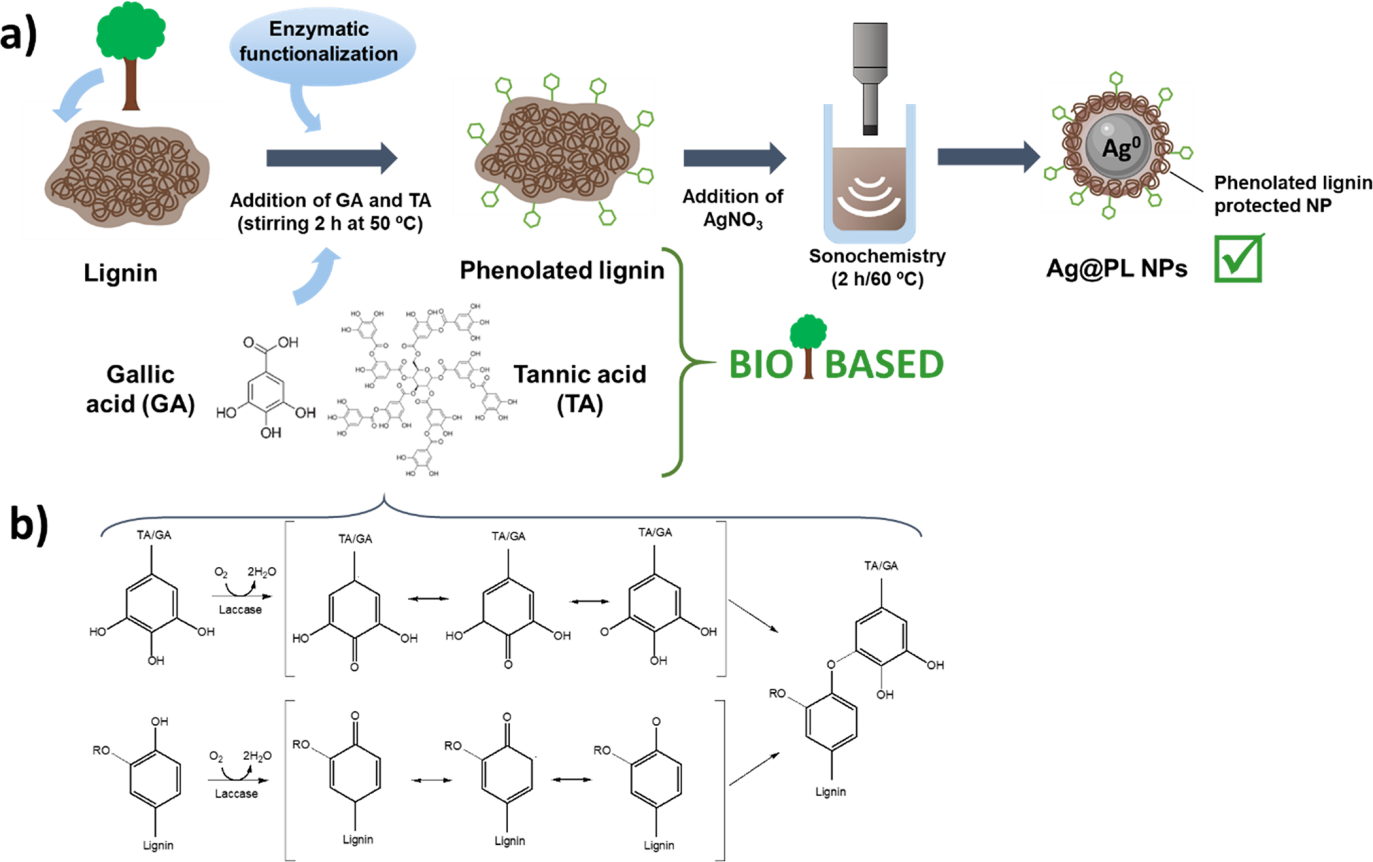
Schematic representation of (a) Ag@PL NP synthesis and disaggregation of particles with sonochemistry technology before incorporation to PICN scaffolds and (b) simplified chemical reactions between gallic and tannic acids and lignin to obtain phenolated lignin compounds.

Ag@PL NPs were synthesized using phenolated lignin to reduce silver ions as can be seen in Figure 1a.^34,35^ Lignin was enzymatically phenolated with tannic acid and gallic acid using the laccase/mediator method. Figure 1b outlines the reaction between the phenolic compounds and lignin. The phenolic content of lignin was analyzed spectrophotometrically. Briefly, the resulting PL was dissolved in water (10 g·L^–1^), and the pH was adjusted to 8 with 1 M NaOH. Afterward, the solution was mixed with 4 mg·mL^–1^ AgNO_3_ (lignin:silver ratio = 3:2) and sonicated at 60 °C for 2 h and 50% amplitude (Sonics and Materials Instrument, Ti-horn, 20 kHz). The NPs were purified by centrifugation at 18,000*g* for 40 min. The nonreacted lignin molecules were removed by centrifuging at 500*g* for 10 min, and the resulting pellet was resuspended in deionized water. The disaggregation of NPs was achieved by low-intensity ultrasonication before usage. More experimental details about this synthesis can be consulted in our previous work.^35^

### 2.3 Deposition of Ag@PL NPs in 3D-Printed PICN Scaffolds (Ag@PL NPs/PICN)

**Figure 2:**
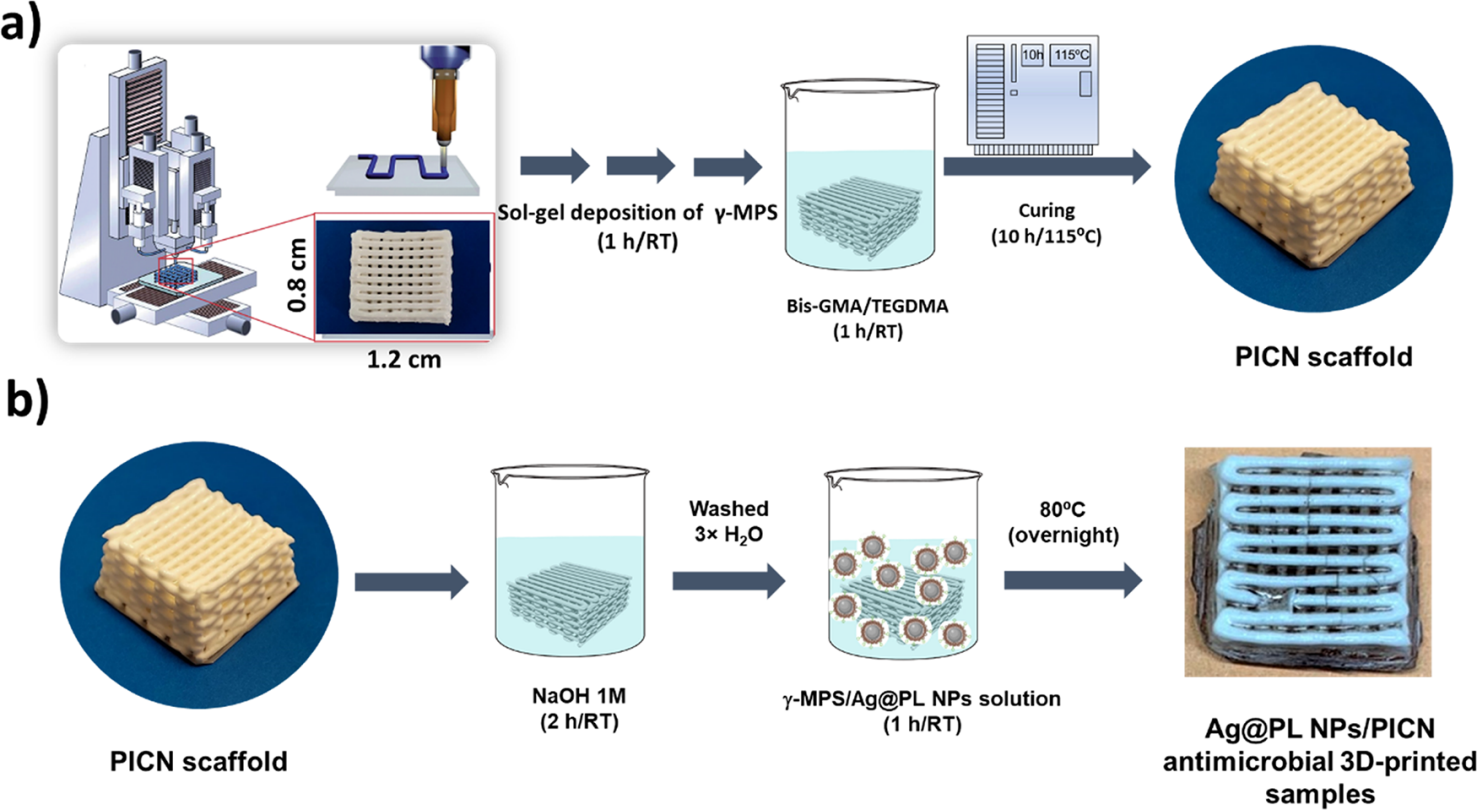
Illustration of the design and fabrication process of antimicrobial 3D-printed PICN samples: (a) 3D-printing of 3Y-TZP filaments with 50% of macropores infilled with the Bis-GMA/TEGDMA copolymer to generate the filled cubic PICN shape and (b) PICN surface activation with NaOH and posterior adsorption of Ag@PL antimicrobial NPs promoted by sol–gel synthesis (γ-MPS in ethanol:H_2_O solution). Printer design represented in panel (a) was reproduced with permissions from refs (40) and (41). Copyrights 2002 and 2005, respectively, WILEY-VCH.

The detailed procedure of 3D-printing of highly porous zirconia (PICN) scaffolds with a 3D Dima Elite dispenser (Nordson Dima, Netherlands) provided with DimaSoft CAD/CAM software and their impregnation with the methacrylate copolymer (Bis-GMA/TEGDMA) were described in our previous work.^9^Figure 2a summarizes such a procedure. After the choice of the properly infiltrated 3D samples, PICN scaffolds were superficially activated by dip-coating in an aqueous solution of NaOH (1 M) for 2 h at room temperature (r.t.), creating hydroxyls and carboxylate groups for a further anchoring of Ag@PL NPs (Figure 2b). Then, those samples were washed three times with distilled water and immediately moved to another vessel containing γ-MPS/Ag@PL NP solution (24 mmol of liquid silane in 100 mL of 3:1 ethanol:Ag@PL NP water solution (2.2 μg·mL^–1^, volume ratio)). The solution was stirred with a magnetic stirrer for 1 h at room temperature before 1 h-long PICN immersion. PICN samples were then moved to an oven (80 °C, overnight) for curing. The absence of particle agglomeration and the homogeneous distribution over the polymer and the zirconia filaments were checked by optical microscopy (OLYMPUS BX51).

### 2.4 Characterization Techniques

Spectroscopy techniques were used for the chemical characterization of the different steps of obtaining Ag@PL NPs/PICN. Fourier transform infrared spectroscopy (FTIR) analysis was performed to distinguish the main absorption bands of functionalized surfaces (Jasco 4100 spectrophotometer). The spectrophotometer is equipped with an attenuated total reflection accessory with a diamond crystal (Specac model MKII Golden Gate Heated Single Reflection Diamond ATR). In total, 64 scans in the range between 4000 and 600 cm^–1^ were obtained for each sample with a resolution of 4 cm^–1^. The Raman spectra were acquired with a Renishaw dispersive Raman microscope spectrometer (InVia Qontor, GmbH, Germany), and data were analyzed with Renishaw WiRE software. The experimental conditions were as follows: 785 nm excitation source; laser power adjusted to 1%; exposure time of 10 s; three accumulation scans; spectral range of 600–4000 cm^–1^.

The distribution and size of freshly synthesized Ag@PL NPs were evaluated by a Philips TECNAI 10 transmission electron microscope manufactured by Philips Electron Optics (Eindhoven, Holland) at an accelerating voltage of 100 kV. The particle size was measured with ImageJ software from TEM images, and the average particle size was determined based on 100 particle size measurements. The scanning electron micrographs (SEM images) were taken with a focused ion beam microscope (Zeiss Neon40) equipped with an energy-dispersive X-ray analysis (EDX) system. The electron beam energy was fixed to 5 kV. EDX was used to check the presence of Ag atoms on the sample surface. To avoid sample charging problems, the cubic structures were attached to a double-side adhesive carbon disc and sputter-coated with a thin layer of carbon.

X-ray photoelectron spectroscopy (XPS) analysis of survey and high-resolution atoms (C 1s, O 1s, Si 2p, and Ag 3d) was carried out to observe whether the AgNPs were well adhered to the PICN surfaces, *i.e.*, to prove their conjugation with the ceramic-polymeric scaffold. The complete description of the equipment and parameters used in this analysis can be seen elsewhere.^9^

### 2.5 Antibacterial Assays

To assess the antibacterial activity of the Ag@PL NPs/PICN, an adhesion assay toward Gram-positive (*S. aureus*) and Gram-negative (*P. aeruginosa*) bacteria was carried out. The adhesion of bacteria onto PICN without NPs was used as a reference to better observe the antibacterial effect of Ag@PL NPs. Prior to the tests, the materials were sterilized under UV light for 30 min, and sterilized tweezers were used to manipulate the samples during the whole process. The bacteria were grown in NB overnight at 37 °C. A dilution of the inoculum was prepared until the optical density measured at a wavelength of 600 nm (OD_600_) was 0.01 (corresponding to 10^5^–10^6^ CFU/mL). Ag@PL NPs/PICN and PICN samples of 2.0 ± 0.2 g were incubated overnight with 2 mL of bacterial suspension in a 24-well plate at 37 °C. The differences in the weight of the samples were compensated by adjusting the volume of bacterial suspensions. Then, samples were sequentially washed three times by immersion in 2 mL of sterile PBS to remove the non-adhered bacteria. Finally, the samples were immersed in 2 mL of fresh PBS and the bacterial cells were detached from the Ag@PL NPs/PICN by vortexing for 1 min and sonication for 20 min in an ultrasonic bath (SONIC 6MX Ultrasonic bath, 37 kHz). After removing the materials from the bacterial suspensions, the number of bacteria adhered on the Ag@PL NPs/PICN samples was estimated using the dilution method and plate counting, obtaining the number of colony-forming units (CFU). Results are expressed in a logarithm of number of bacteria, log(CFU/mL). The percentage of reduction of adhered bacteria was calculated using PICN as a reference (eq 1):
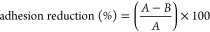
1where *A* is the number of bacteria adhered to PICN, and *B* is the number of bacteria adhered to Ag@PL NPs/PICN. Bacterial suspensions incubated in the absence of the materials, either subjected or not to vortexing and ultrasonication, were used as controls (Figure S1, Supporting Information).

### 2.6 Biocompatibility Assays

The biocompatibility of the Ag@PL NPs/PICN and PICN samples was assessed using an indirect method by growing the cells in a medium that was previously incubated with zirconia samples. Prior to the tests, Ag@PL NPs/PICN and PICN samples (2 g) were incubated in 2 mL of DMEM for 24 h or 7 days at 37 °C. Then, 100 μL of this medium was placed in a 96-well tissue culture-treated polystyrene plate where 6 × 10^4^ cells per well were previously seeded. After incubation at 37 °C in a humidified atmosphere of 5% CO_2_ for 24 h, the medium was withdrawn and the cell viability was assessed by incubating the cells with 100 μL of AlamarBlue (10% v/v in DMEM) for 4 h at 37 °C. The percentage of cell viability was estimated using the fluorescence values (λ_ex_ = 550 nm and λ_em_ = 590 nm) of the wells containing only cells and the AlamarBlue reagent as a reference (growth control). Wells containing only the AlamarBlue reagent were used as the blank group. The percentage of cell viability was estimated as follows:
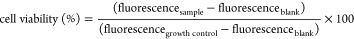
2

More details about the experimental procedure can be found in ref (42).

## 3 Results and Discussion

### 3.1 Functionalization of PICN with Ag@PL NP Antimicrobial Particles

**Figure 3:**
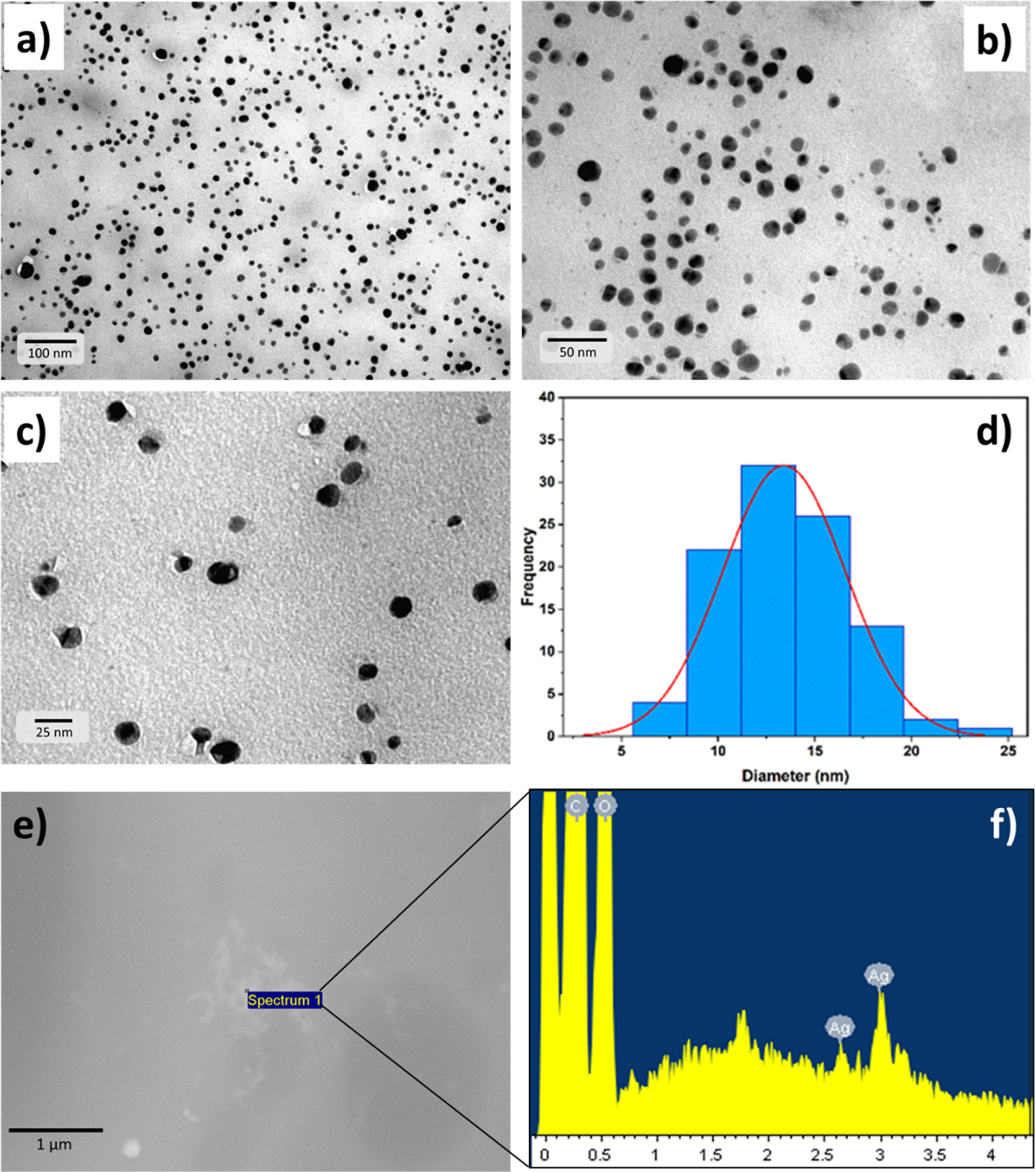
(a–c) TEM images of Ag@PL NPs at different magnifications before PICN attachment, (d) nanoparticle distribution by size and frequency, and (e, f) SEM micrograph and EDX spectrum of aggregated Ag@PL NPs above 3D-printed PICN scaffolds. The nanoparticle distribution and size in TEM were analyzed with ImageJ software, and they were derived from imaging 100 particles.

The complete characterization of AgNPs protected with PL was previously introduced by Tzanov and co-workers.^35^ The Ag@PL NPs used in this work were prepared following the same procedure, and the particle size diameter measured by TEM was similar to that previously reported (13.4 ± 3.2 nm) (Figure 3a–d).

The activation of 3D-printed PICN with Ag@PL NPs was only possible by quenching the copolymer film surface with NaOH (1 M) and subsequently anchoring the protected NPs by using the sol–gel technology, as described in Section 2.3. Other tested methodologies (e.g., plasma activation and a mixture of Ag@PL NPs with Bis-GMA/TEGDMA monomers prior to copolymerization) failed, and no silver atoms could be found on the surface of the cubic structure. Although the small size of the particles makes their observation in the SEM micrographs difficult, Ag atoms were detected by EDX analyses (Figure 3e,f) on the top of the PICN surface after applying the sol–gel technology. Thus, successful adhesion of the bactericide particles was proved by SEM–EDX and, additionally, by optical microscopy (Figure 3).

**Figure 4:**
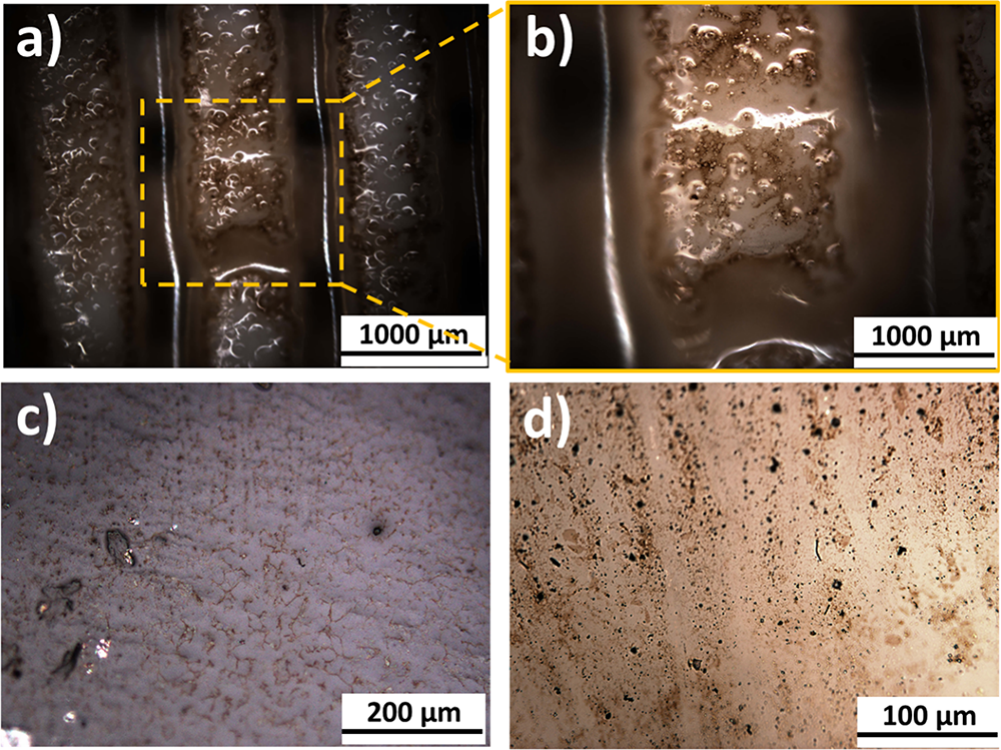
Optical micrographs of the surface of PICN filaments showing the adsorption of the Ag@PL NPs: (a, b) 5× magnification, (c) 20× magnification, and (d) 50× magnification.

As can be seen in Figure 4a,b, the natural roughness of the zirconia filaments facilitates the incorporation of the Ag@PL NPs promoted by the sol–gel mixture. Closer inspection outside the filament top (valleys shown in Figure 4a) revealed also the homogeneous distribution of the Ag@PL NPs inside the methacrylate copolymer film, determined by the dark color particles seen in Figure 4c,d. It is important to highlight that optical microscopy only allows for observation of even distribution of the lignin macromolecules based on what the homogeneity of Ag@Pl NP distribution was expected.

The high roughness of the 3Y-TZP filaments, after the sintering process at high temperatures, prevents the correct measurement of this property, and there are also difficulties in the measurements of the hydrophobicity/hydrophilicity properties of the film before and after the NP incorporation.

**Figure 5:**
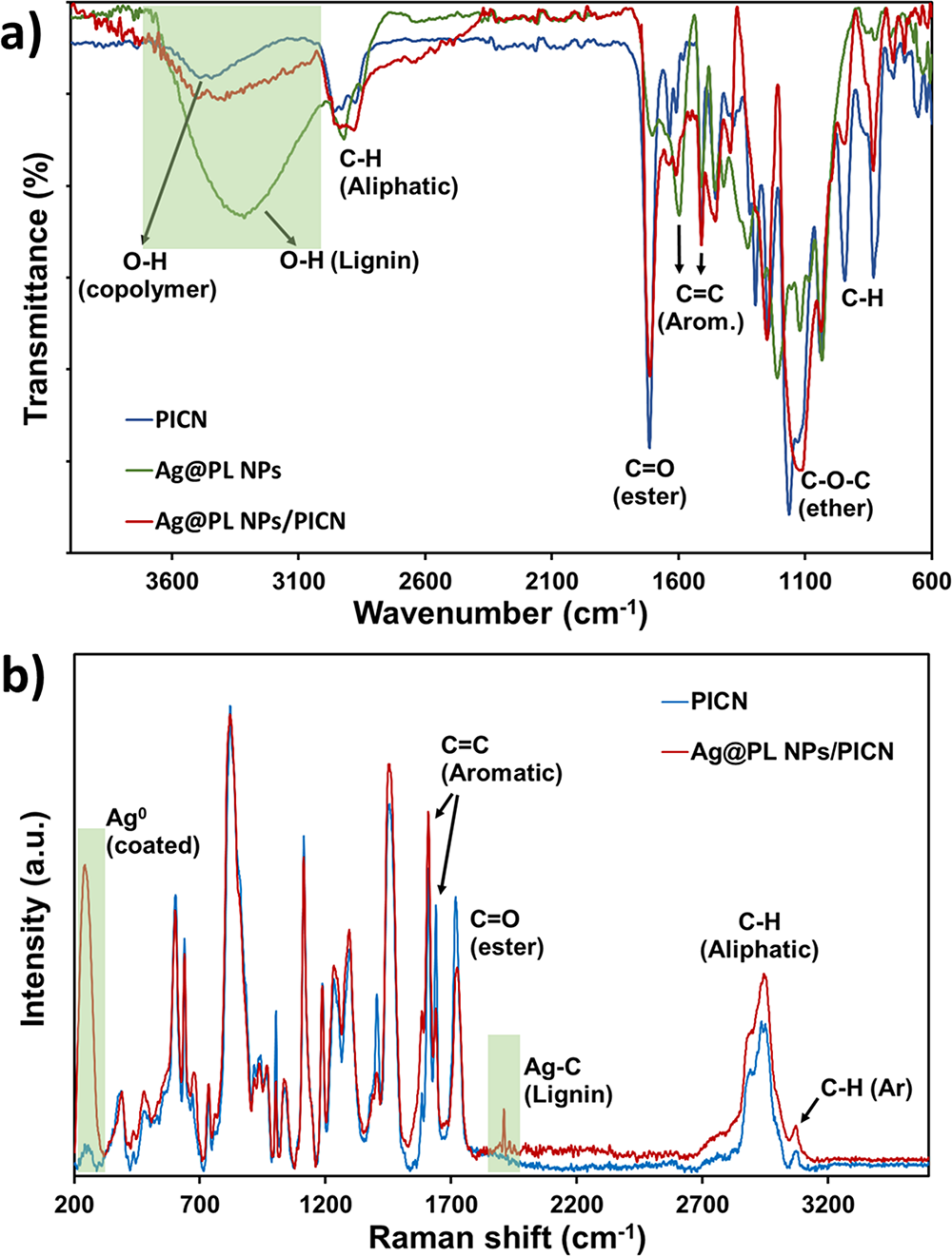
(a) FTIR spectra of 3D-printed PICN, dry Ag@PL NP, and Ag@PL NPs/PICN samples. (b) Raman spectra of PICN and Ag@PL NPs/PICN. The most relevant absorption bands are highlighted in both cases.

The characterization of the hybrid material was not an easy task because both the adhesive (acrylate copolymer) and the AgNPs have organic groups that are very similar in their structures (alcohol, aromatic, aliphatic, and ethers). Lignin is a complex chemical compound constituting up to a third of the dry mass of plants depending on the species. This natural biopolymer has a high content of aromatic rings and hydroxyl groups, which were evidenced in the FTIR spectra (Figure 5a). The broad and intense band at ∼3400 cm^–1^ in Ag@PL NPs corresponds to the high number of hydroxyl groups in the lignin structure. The broadening of such an absorption band on the surface of 3D-printed Ag@PL NPs/PICN samples proves the successful incorporation of the NPs promoted by alkali activation. Moreover, C=C vibrations from aromatic rings of both the Bis-GMA monomer (copolymer) and lignin are reflected by sharp bands at ∼1600 and 1509 cm^–1^. The peak at 2920 cm^–1^ also includes methylene and methyl bonds of the copolymer and the nanoparticles. However, the most relevant absorption bands that were identified, also in the hybrid material (Ag@PL NPs/PICN), are those associated with the presence of ester and ether linkages at 1712 cm^–1^ (C=O) and at 1100–1160 cm^–1^ (C–O), respectively, from Bis-GMA and TEGDMA units and also phenolated lignin. Lignin contains C=O groups from unconjugated carbonyl groups (∼1705–1720 cm^–1^), and in phenolated lignin, the intensity of this peak increases due to the presence of tannic acid and gallic acid, which also have C=O groups.^43−47^

The high density of organic groups with low polarity (C=C and =C—H) led us to use Raman spectroscopy to ascertain the presence of other linkages. Figure 5b represents the Raman spectra for PICN and PICN modified with Ag@PL NPs. Clearly, the absorption bands of C=C aromatic bonds (∼1600 cm^–1^) are more intense than that of the C=O group (1729 cm^–1^), while C–H (Ar) is clearly observed at 3072 cm^–1^ due to the complete absence of hydroxyl absorption bands.^48,49^ Moreover, silver nanoparticle lattice vibrational modes are also identified at 250 cm^–1^ and at ∼1900 cm^–1^ in the region of metal carbonyls, which would suggest an interaction between AgNPs and the lignin matrix and the stability of the complex.^50,51^

**Figure 6:**
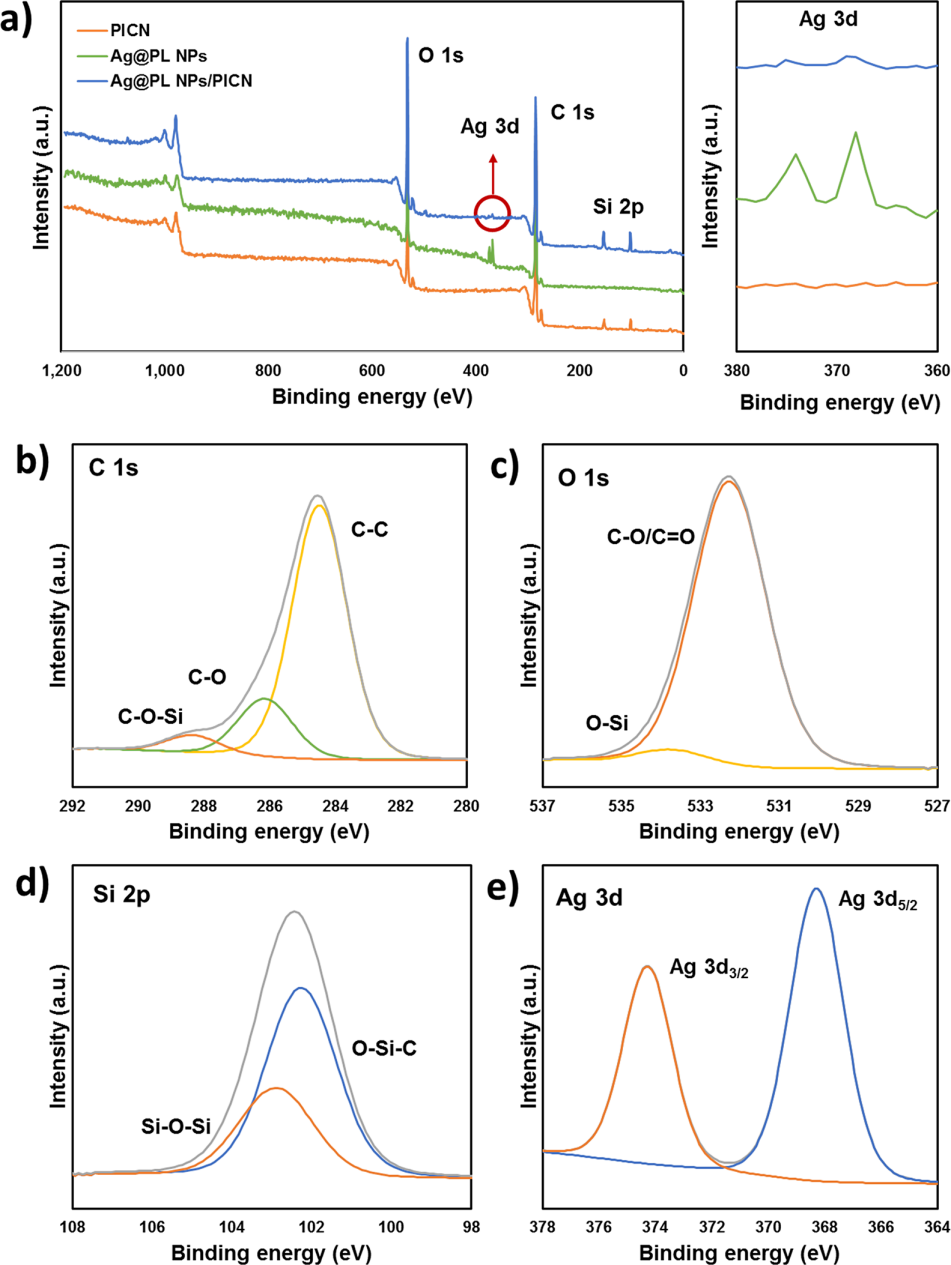
(a) XPS survey spectra of PICN, dry Ag@PL NP, and Ag@PL NPs/PICN samples. (b–e) High-resolution spectra of the Ag@PL NPs/PICN sample: C 1s (b), O 1s (c), Si 2p (d), and Ag 3d (e).

Although the spectroscopy characterization confirms the well-assembled AgNPs to the methacrylate adhesive, the nature of such bonding interactions can only be approached by XPS. The survey spectra (Figure 6a) show atoms of C 1s (∼285 eV) and O 1s (∼530 eV) for all samples, whereas those of Ag 3d (374 and 368 eV) are only present in the pure Ag@PL NP and Ag@PL NPs/PICN samples. Moreover, the Si 2p binding energies (102 eV) are identified either in PICN or the PICN surface modified with the antibacterial particles, as expected, since the copolymer infiltration to the pores of 3D-printed zirconia is also optimized by the sol–gel technology.^9^ Particularly interesting is the absence of Zr 3d atoms (183 eV) in the survey spectrum of PICN, belonging to the ceramic structure, confirming the well-covered surface of all samples. The high-resolution spectra (Figure 6b–e) from C, O, and Si elements confirmed the covalent bonding nature of such atoms with the help of a silanization reaction. Therefore, after silanization, C–O–Si (288 eV, Figure 6b), O–Si (531 eV, Figure 6c), and Si–O–Si and Si–O–C (103 and 102 eV, Figure 6d) appear on the PICN-functionalized surface. Furthermore, the incorporation of Ag@PL NPs in PICN scaffolds is able to maintain the active metallic condition after the sol–gel process of application, showing similar values of binding energies related to the Ag element (374 and 368 eV for Ag 3d_3/2_ and Ag 3d_5/2_, respectively) (Figure 6e).

Afterward, the evaluation of the antimicrobial activity and biocompatibility of the whole system was carried out with *S. aureus* and *P. aeruginosa* bacteria and with two cell lines, keratinocytes and fibroblasts.

### 3.2 Effect of the Presence of Ag@PL NPs on the Antimicrobial Properties of PICN Scaffolds

**Figure 7:**
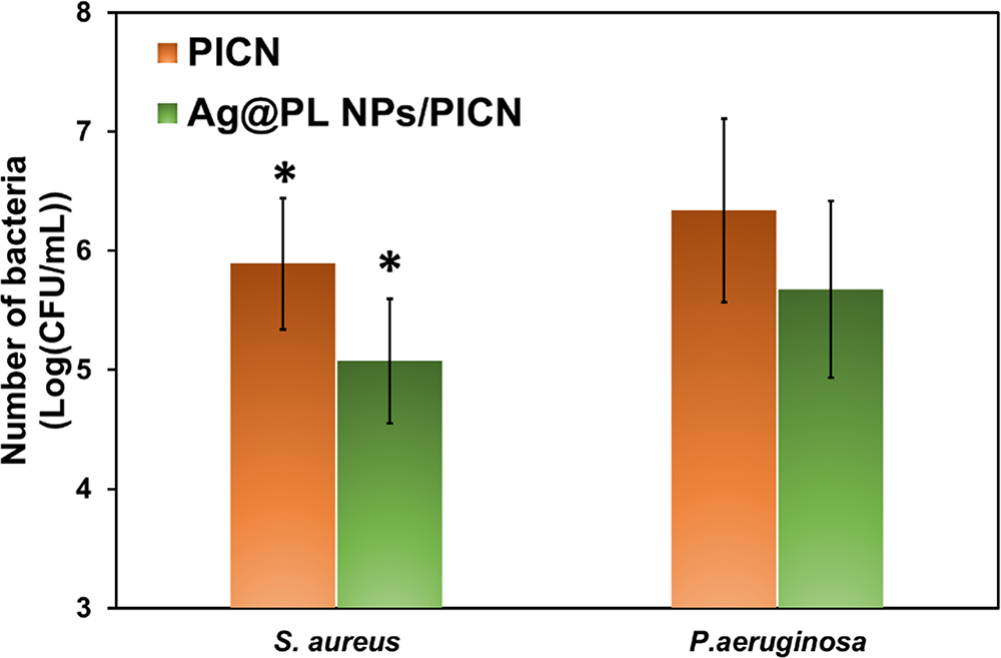
Number of bacteria (*S. aureus* and *P. aeruginosa*) adhered onto 3D-printed PICN and Ag@PL NPs/PICN scaffolds, expressed in the logarithm of viable bacteria, log(CFU/mL). Results marked with stars refer to the confidence level where *p* < 0.05 using Student’s *t*-test. The log (CFU/mL) values can be consulted in Table S1 (Supporting Information).

There are about 5 billion bacteria in a human oral cavity. The antimicrobial effects of silver nanoparticles are well known.^52,53^ If PICN scaffolds are intended for future dentistry applications, which was the focus of the research at its preliminary stage, the antibacterial activity of the Ag@PL NPs/PICN should be assessed. For this study, two clinically relevant pathogens (the Gram-positive *S. aureus* and the Gram-negative *P. aeruginosa*), also present in our oral cavity, were chosen. The initial antibacterial activity was assessed by counting the number of bacteria adhered onto the surface of Ag@PL NPs/PICN in comparison to that adhered onto PICN surfaces (used as a control) (Figure 7 and Table S1 in the Supporting Information). The number of *S. aureus* adhered onto nonfunctionalized PICN was 5.89 ± 0.55 log, while it was reduced to 5.07 ± 0.52 log for Ag@PL NPs/PICN, corresponding to about 90% reduction of bacteria adhered. In the case of *P. aeruginosa*, the number of bacteria decreased from 6.34 ± 0.77 log to 5.68 ± 0.74 log, which corresponds to about 73% reduction with respect to the nonfunctionalized PICN surface. Both percentages were calculated by using eq 1 (Section 2.5). The antibacterial effect of AgNPs is attributed to both their attachment to the bacterial cell and the release of Ag^+^ ions. Some studies showed that AgNPs were more effective against Gram-negative bacteria, which was ascribed to their thinner peptidoglycan layer in comparison with Gram-positive bacteria.^52−54^ In the present work, Ag@PL NPs/PICN were more effective against the Gram-positive bacteria. It should be noted that we have reported higher adhesion of Gram-negative bacteria to PICN scaffolds (without Ag@PL NPs) in our previous study.^9^ Moreover, Ag@PL NPs incorporated into polyurethane (PU) foams exhibit high antibacterial activity against both bacterial lines, reaching over 4.6 and 5.6 log reduction, respectively, for *P. aeruginosa* and *S. aureus*.^35^ Their higher antibacterial capacity in the foam materials in comparison with Ag@PL NPs/PICN hybrid materials may be due to their different NP loads. To obtain antibacterial PICN materials, we chose a Ag@PL NP concentration based on our previous work, in which the totality of the NPs was incorporated in the foam. However, the efficiency of the deposition of Ag@PL NPs was not 100%, so the final content of NPs in the materials was lower than expected. This may explain the lower antibacterial activity of PICN scaffolds in comparison with the PU foams.

### 3.3 Effect of the Presence of Ag@PL NPs on the Biocompatibility Properties of PICN Scaffolds

**Figure 8:**
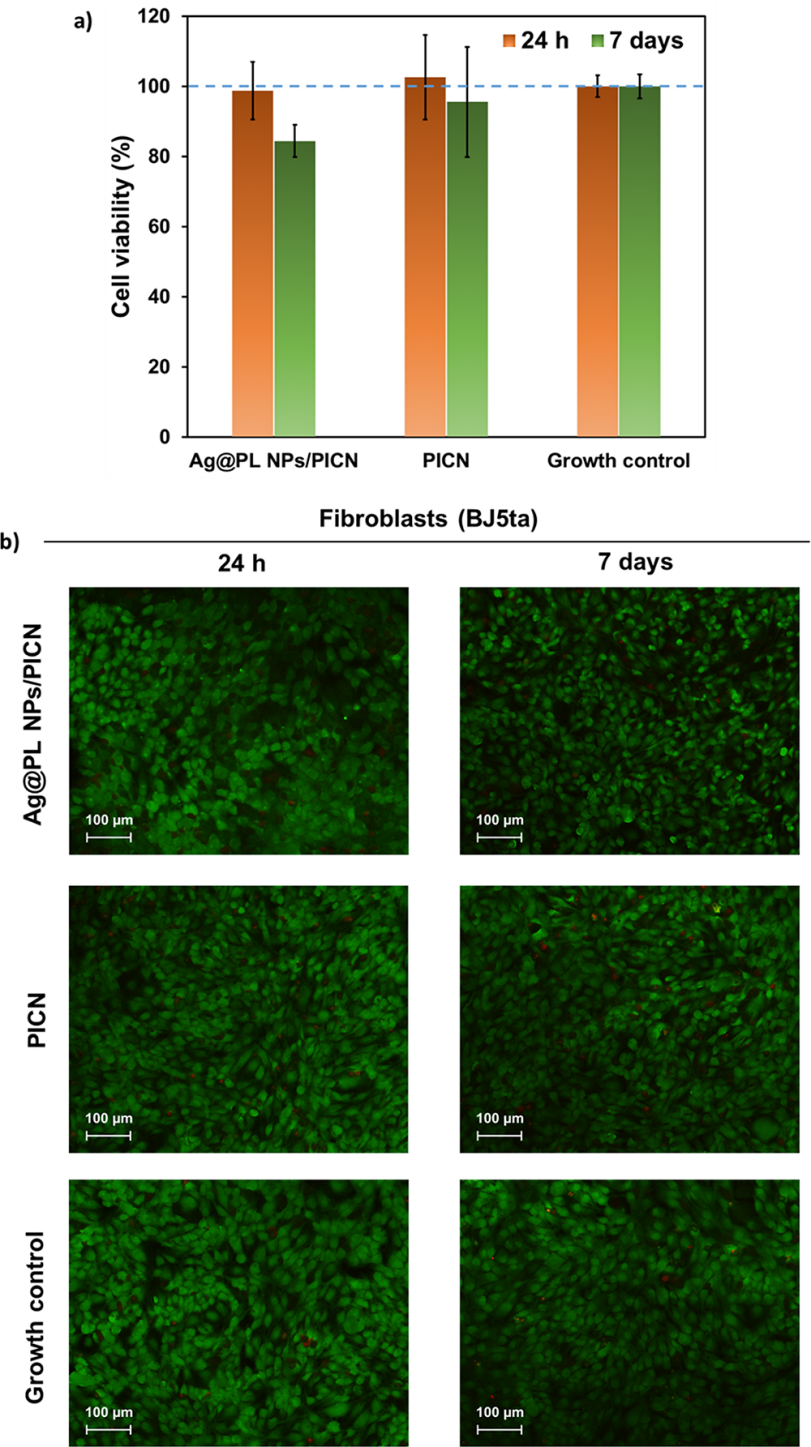
(a) Cell viability and proliferation (%) of human fibroblast-like cells (BJ5ta) incubated with medium previously exposed to Ag@PL NPs/PICN and PICN samples for 24 h or 7 days. The control is related to the media without the 3D-printed pieces. (b) Microscopy images of live/death assay of human fibroblasts incubated with medium exposed to Ag@PL NPs/PICN and PICN for 24 h and 7 days. The assay reagent (AlamarBlue) stains the live cells in green and the dead ones in red. One representative image of each experimental group (three replicates) was chosen. Growth control refers to cells incubated with fresh medium (Figure S1).

**Figure 9:**
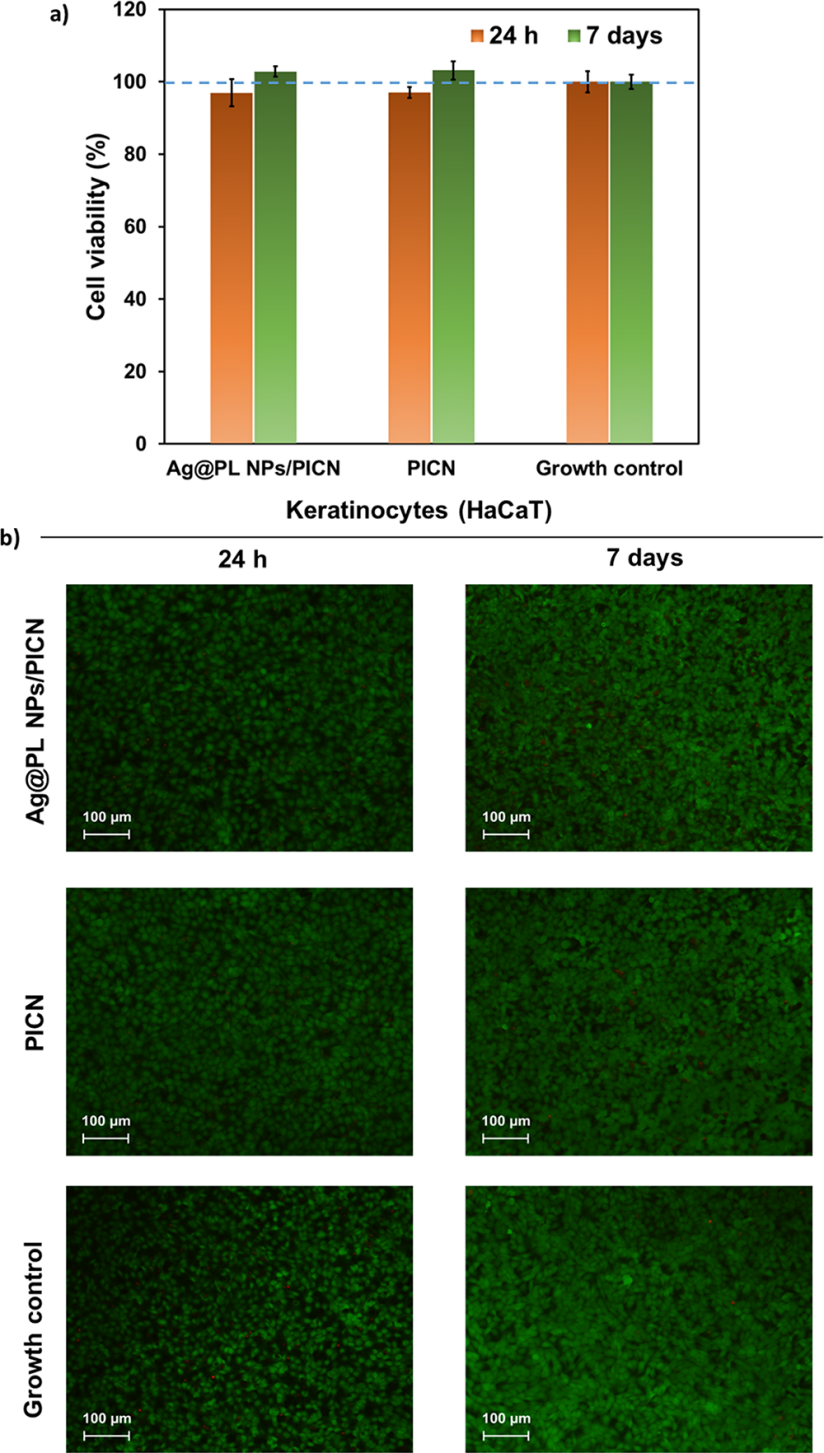
(a) Cell viability and proliferation (%) of human keratinocyte cells (HaCaT) incubated with medium previously exposed to Ag@PL NPs/PICN and PICN samples for 24 h or 7 days. The control is related to the media without the 3D-printed pieces. (b) Microscopy images of live/death assay of human fibroblasts and keratinocytes incubated with medium exposed to Ag@PL NPs/PICN and PICN for 24 h and 7 days. The assay reagent (AlamarBlue) stains the live cells in green and the dead ones in red. One representative image of each experimental group (three replicates) was chosen. Growth control refers to cells incubated with fresh medium (Figure S1).

The biocompatibility of implants is a crucial criterion for their biomedical application. In the case of silver-containing implants, the release of Ag may cause cytotoxicity, which is attributed to the generation of ROS, destabilization of the cell membrane, and inactivation of essential enzymes.^55,56^ The cell viability of pure PICN and Ag@PL NPs/PICN samples was assessed *in vitro* employing two different cell models, HaCaT and BJ5ta, which are immortalized cell lines from adult human skin with keratinocyte and fibroblast-like morphology, respectively. The culture media previously pre-incubated with Ag@PL NPs/PICN and PICN samples for 24 h or 7 days were used to grow the cells for 24 h, while a fresh medium that has not been in contact with the samples was used to grow control cells. Figures 8a and 9a display quantitative results, which correspond to the average of three independent replicas for each system, and they are expressed in terms of cell viability relative to control cells. Furthermore, the whole procedure for the cell viability study and the microscopy images showing the cells stained with AlamarBlue can be seen in Figure S1 (Supporting Information) and Figures 8b and 9b, respectively.

As shown, the number of viable cells is similar to that of the control for both cell lines (BJ5ta and HaCaT). This behavior was maintained for Ag@PL NPs/PICN, indicating that the functionalization with Ag@PL NPs does not have a major impact on the viability of the cells. Even after 7 days, the media incubated with Ag@PL NPs/PICN only slightly decreased the viability of the cell lines down to 97% in the case of fibroblast cells (Figure 8a). After 7 days of incubation of Ag@PL NPs/PICN in medium, a certain amount of Ag@PL NPs was probably released from the scaffold to the medium. Once in contact with the fibroblast cells, the released NPs slightly affected the cell viability (reduction to 84%), as can be seen in the same plot. Meanwhile, the viability of keratinocyte cells (HaCaT) was slightly higher for both Ag@PL NPs/PICN and PICN than for the control (Figure 9a). Therefore, the opposite behavior was found for keratinocyte cells. On the other hand, the fact that HaCaT cells systematically exhibit higher proliferation than BJ5ta cells has been attributed to the high capacity of the former to differentiate and proliferate *in vitro.*(57)

Overall, cell viability results for both PICN and Ag@PL NPs/PICN samples were higher than 80% in all cases, independent of the cell line, which is an acceptable value for biomedical applications.^58−60^ The high cell viability values found did not decrease when PICN scaffolds were functionalized with antimicrobial NPs, indicating that neither PICN nor Ag@PL NPs induce cytotoxic effects *in vitro* against keratinocytes and fibroblast-like cells. This important conclusion is supported by representative microscopy images of BJ5ta and HaCaT, in which we can appreciate the cell growth (Figures 8b and 9b). As can be seen, the live/dead staining represented in the images is consistent with the viabilities displayed in Figures 8a and 9a, and most of cells remain alive after 24 h and 7 days of incubation. Thus, the high cell viability has been associated with the biocompatibility of the control and studied substrates.

## 4 Conclusions

In the present work, a successful reduction of Gram-positive and Gram-negative bacteria above the PICN surfaces functionalized with silver nanoparticles has been achieved after 24 h of microorganism incubation. It was attributed to the effective stabilization of such NPs on the complex 3D structure. Enzymatically phenolated lignin has been used as a reducing agent to obtain stable and biocompatible silver NPs. The challenge of attaching Ag@PL NPs to the surface has been overcome by combining the Ag@PL NPs with silane (γ-MPS) as a coupling agent between the zirconia surface and the polymer structure. The permanent attachment of the NPs has been proven by multiple techniques described above, including X-ray photoelectron spectroscopy, where a clear peak of Ag 3d binding energy appeared in the functionalized samples. Moreover, the results obtained show the capacity of the Ag@PL NPs/PICN structure to avoid the adhesion of bacteria onto their surfaces (over 24 h), which is attributed to the bactericidal effect of silver in the form of NPs, without the detriment of the viability of human cell lines. In this case, either fibroblast or keratinocyte cells had about of 80–97% of compatibility after 7 days of incubation, therefore demonstrating that any amount of metal NPs released in these media, over this period (24 h and 7 days), is not toxic for the cell adhesion and proliferation. Taking into account that biomedical implants must have much longer stability than assayed, future works driven to investigate the antimicrobial property of Ag@PL NPs in PICN scaffolds in a long term of bacteria incubation are envisaged to validate these lab-proof results.

## Supporting Information Available

Table S1: values of number of bacteria in log(CFU/mL) adhered to PICN and Ag@PL NPs/PICN; Figure S1: schematic representation of the procedure carried out to apply the indirect method used in this work to explore the cell viability of PICN scaffolds (PDF)
