# Supplementary material for: 3D-Printed Polymer-Infiltrated Ceramic Network with Antibacterial Biobased Silver Nanoparticles
Source: ACS Appl Bio Mater. 2022 Sep 27;5(10):4803–13. doi: 10.1021/acsabm.2c00509 (PMC9923783; doi:10.1021/acsabm.2c00509)
Supplement: Supplementary file 1 — mt2c00509_si_001.pdf [file mt2c00509_si_001.pdf]

3D-printed polymer-infiltrated ceramic network with antibacterial bio-based silver nanoparticles

Ľudmila Hodásová,[a,b,d] A. Gala Morena,[c] Tzanko Tzanov,[c] Gemma Fargas,[b,d] Luis Llanes,[b,d] Carlos Alemán[a,b,e] and Elaine Armelin*[a,b]

[a] *Departament d'Enginyeria Química, IMEM-BRT, EEBE, Universitat Politècnica de Catalunya, C/ Eduard Maristany, 10-14, Ed. I, 2nd floor, 08019, Barcelona, Spain.*
[b] *Barcelona Research Center in Multiscale Science and Engineering, Universitat Politècnica de Catalunya, C/ Eduard Maristany, 10-14, basement S-1, 08019, Barcelona, Spain.*
[c] *Grup de Biotecnologia Molecular i Industrial, Department of Chemical Engineering, Universitat Politècnica de Catalunya, Terrassa, 08222, Spain.*
[d] *Departament de Ciència i Enginyeria de Materials, CIEFMA, EEBE, Universitat Politècnica de Catalunya, Campus Diagonal Besòs –, C/ Eduard Maristany, 10-14, Building I, 1st floor, 08019, Barcelona, Spain.*
[e] *Institute for Bioengineering of Catalonia (IBEC), The Barcelona Institute of Science and Technology, Baldiri Reixac 10-12, 08028, Barcelona, Spain.*

*Corresponding author: E-mail: elaine.armelin@upc.edu (Elaine Armelin)

Table S1. Number of bacteria in log(CFU/mL) adhered to PICN and Ag@PL NPs/PICN. Control refers to bacteria incubated in absence of the materials.

	S. aureus	*P. aeruginosa*
PICN	5.89	6.34
Ag@PL NPs/PICN	5.07	5.68
Control (with ultrasound)	5.81	6.75
Control (without ultrasound)	8.87	6.61

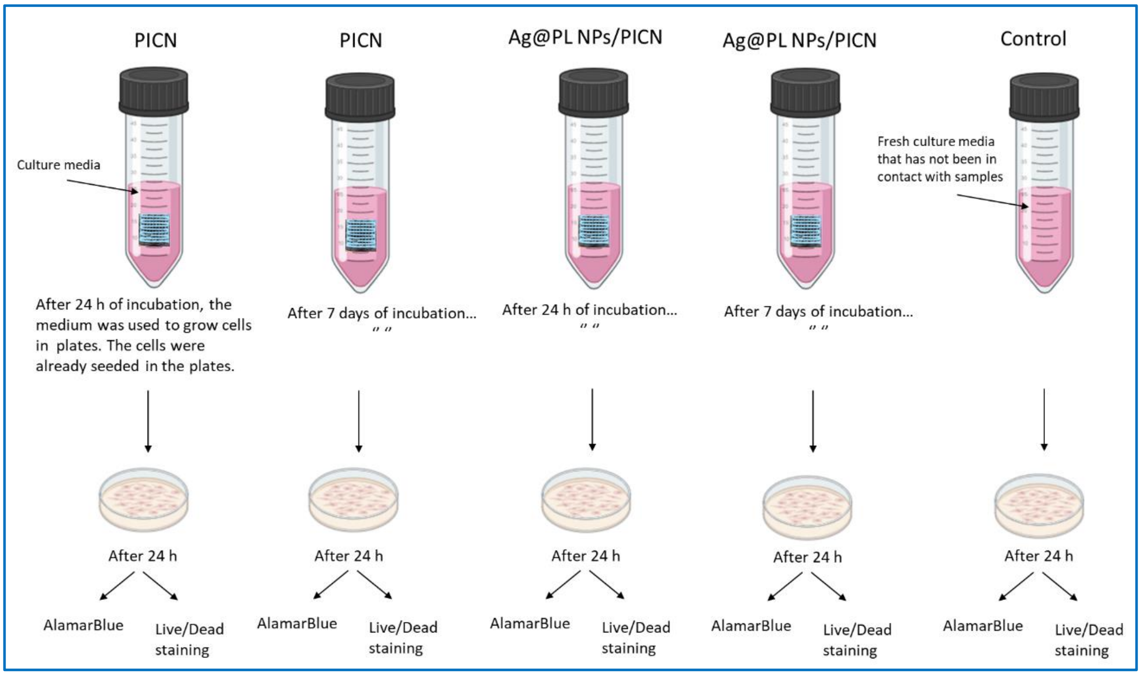

Figure S1. Representation of the indirect method used in this work to explore the cell viability of PICN scaffolds, compared to the control media (without PICN samples).
